# Using chimeric rice proteins to make heads or tails of the function of repetitive elements in Gγ subunits

**DOI:** 10.1093/plcell/koaf080

**Published:** 2025-04-03

**Authors:** Julie Robinson

**Affiliations:** Assistant Features Editor, The Plant Cell, American Society of Plant Biologists; HudsonAlpha Institute for Biotechnology, Huntsville, AL 35806, USA

Many animals have tails that function to maintain balance while they're stalking through forests, scurrying along tree branches, or leaping onto your kitchen counter. Monkeys, tigers, squirrels, and mice all have tails that serve this purpose and have the same general structure—vertebrae surrounded by muscle—but also have structural differences that optimize balance for each animal according to the different ways they move. Some tails even serve additional functions, such as grasping objects or communicating. Similarly, proteins with conserved structural elements often serve similar purposes but in different ways or to different degrees. This article will discuss the variable ends of a different type of tail on a protein that regulates rice grain size.

G proteins are conserved in all eukaryotes and have a multitude of functions in plants, such as regulating growth and stress responses by translating environmental signals into physiological responses so that plants can respond accordingly to environmental changes and challenges (Lee 2024). Heterotrimeric G proteins contain Gα, Gβ, and Gγ subunits. In rice, overexpressing any 1 of 3 Gγ subunits—GS3, GGC2, or DEP1—affects grain size (Sun et al. 2018). All 3 share head domains with high sequence similarity at the N terminus, and GGC2 and DEP1 share tail domains at the C terminus that contain many of the same repetitive elements. Interestingly, GS3 has a negative effect on grain size while GGC2 and DEP1 have a positive effect, suggesting that the tail domains of GGC2 and DEP1 are responsible for increasing grain size. In new work, **Shengyuan Sun and colleagues (Sun et al. 2025)** seek to identify the specific elements in Gγ subunit tail domains that affect grain size in rice and further aim to determine their evolutionary lineages.

In molecular genetics, an excellent way to determine the function of a gene, protein, or parts thereof is to manipulate them and observe for changes in function. In this case, what would be the effect of making a chimeric protein that contains the head domain of GS3 and the tail domain of DEP1? Sun and colleagues answered this question, creating different combinations of the head and tail domains of GS3, GGC2, and DEP1. They found that overexpressing a chimeric protein composed of the GS3 head domain and a tail domain from either GGC2 or DEP1 resulted in larger grain sizes, similar to those observed when overexpressing wild-type GGC2 or DEP1 (Figure A). Conversely, combining the tail domain of GS3 with the head domain of either GGC2 or DEP1 slightly reduced grain size, thus confirming that it is indeed the conserved tail domain of GGC2 and DEP1 that is responsible for the positive effect on grain size (Figure A). The researchers further sought to show whether the repetitive elements within the tail domains are responsible for the tails' effect on grain size. By systematically deleting different sections of repetitive elements in different combinations, Sun and colleagues determined that elements I-II and the L motif are responsible for positively regulating grain size in rice and do so additively (Figure B).

**Figure.**
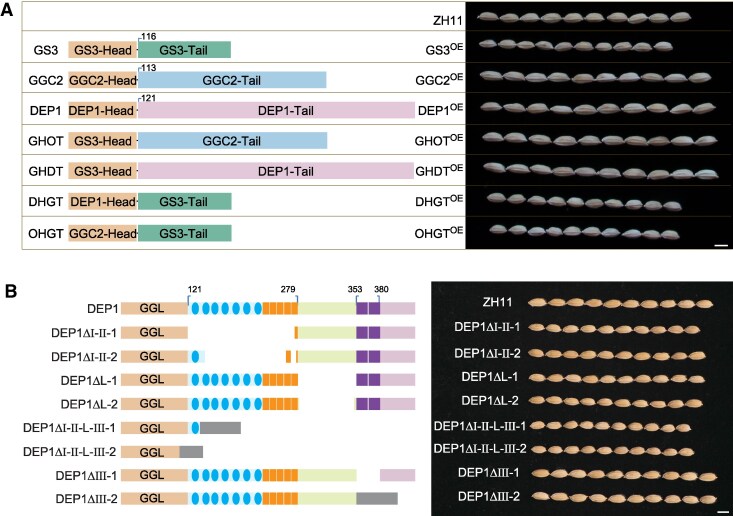
**A)** Overexpressing chimeric Gγ proteins in rice (left) yields grains (right) that are significantly different sizes depending on the tail that is attached. **B)** Systematic deletion of different sections of repetitive elements reveals the importance of elements I-II (blue and orange) and the L motif (green). ZH11 is the wild type. Adapted from Sun et al. (2025), Figures 1, 3.

Next, Sun and colleagues asked: How conserved is the function of these Gγ protein repetitive elements in monocots? To investigate this question, the authors tested the function of the tail domains of 4 corn Gγ proteins that are homologs to rice GS3, GGC2, and DEP1, containing similar numbers of the same repetitive elements in their tail domains. Mutating the GGC2 and DEP1 homologs via CRISPR/Cas9 indeed reduced corn kernel size, as did overexpressing GS3, suggesting a conserved role of these tail domains between corn and rice. Furthermore, Sun and colleagues performed a proximate collinearity analysis that revealed DEP1 to be the evolutionarily oldest tailed Gγ protein in monocots, which gave rise to GGC2 and GS3 in grasses after multiple genomic duplications.

The findings presented in this paper not only reveal novel repetitive elements and their physiological effects, but they also have implications for improving agricultural output by demonstrating the generation of chimeric proteins as a method of improving crop yields.

## Recent related articles in *The Plant Cell*

- Xie et al. (2024) revealed regulation of grain size and weight in rice via E3 ligase ubiquitination of an NAC transcription factor.
- Kim et al. (2024) used chimeras of PIF1 and PIF4 to study their functional diversification in Arabidopsis.
- Chen et al. (2024) showed that OsMOB1A, OsSTK38, and cyclin C function in a molecular pathway regulating grain size and weight in rice.

## Data Availability

There are no new data associated with this article.

## Dive Curated Terms

The following phenotypic, genotypic, and functional terms are of significance to the work described in this paper:

DEP1 Gramene: Os09g0441900
DEP1 Araport: Os09g0441900

GGC2 Gramene: Os08g0456600
GGC2 Araport: Os08g0456600
